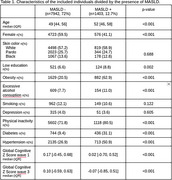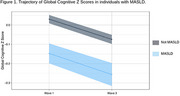# Association of Metabolic dysfunction‐associated steatotic liver disease, MASLD, with Cognitive Function in a Brazilian Cohort

**DOI:** 10.1002/alz70860_101804

**Published:** 2025-12-23

**Authors:** Luis Gustavo Sampaio, Raphael Machado Castilhos, Bruce Duncan, Maria Inês Schmidt, Natan Feter

**Affiliations:** ^1^ Hospital de Clínicas de Porto Alegre, Porto Alegre, Rio Grande do Sul, Brazil; ^2^ Universidade Federal do Rio Grande do Sul, Porto Ale, Rio Grande do Sul, Brazil; ^3^ Hospital de Clinicas de Porto Alegre, Porto Alegre, Rio Grande do Sul, Brazil; ^4^ Universidade Federal do Rio Grande do Sul, Porto Alegre, Rio Grande do Sul, Brazil; ^5^ Postgraduate Program in Epidemiology, Universidade Federal do Rio Grande do Sul, Porto Alegre, Brazil; ^6^ Medical School, Universidade Federal do Rio Grande do Sul, Porto Alegre, Brazil; ^7^ Universidade Federal do Rio Grande do Sul, Porto Alegre, Brazil

## Abstract

**Background:**

Metabolic dysfunction‐associated steatotic liver disease (MASLD) has recently been studied as a potential risk factor for cognitive decline (CD). However, the evidence in the literature shows conflicting results. We aim to evaluate MASLD as a risk factor for cognitive decline in the Estudo Longitudinal da Saúde do Adulto (ELSA‐Brasil).

**Methods:**

Risk factors, sociodemographic variables, and MASLD were collected between 2008 and 2010 (wave 1) and the global cognitive function score was established, by averaging Z scores from six standardized tests, approximately 8 years later (wave 3). Steatosis was diagnosed by ultrasonography. MASLD was defined as steatosis with at least one of five factors: obesity, diabetes (or pre‐diabetes), hypertension, high triglycerides, or low high‐density cholesterol. The effect of the MASLD on cognitive decline between the two waves was assessed with a mixed linear model (MLM). All models were adjusted for age, sex, and dementia risk factors (low education, physical inactivity, depression, alcohol and tobacco consumption).

**Results:**

Of the 15,105 individuals of the ELSA‐Brasil, 11,032 (73%) had all variables and were included in the analysis. Of these, 1403 (12.7%) had MASLD. Individuals with MASLD were older (52 [46‐58] vs 49 [44‐56]), more frequently male (58.9% vs 40.5%), and more often presented dementia risk factors (hypertension, diabetes, obesity, physical inactivity, and excessive alcohol consumption) (Table 1). In the MLM analysis, controlling for the confounders, participants with MASLD began with a 0.15 lower z‐score and presented a more rapid decline (‐0.06 z‐scores in 8 years; *p* <0.0001) (Figure 1).

**Conclusions:**

MASLD was associated with cognitive decline in the ELSA‐Brasil study.